# Mucociliary dysfunction in HIV and smoked substance abuse

**DOI:** 10.3389/fmicb.2015.01052

**Published:** 2015-10-14

**Authors:** Srinivasan Chinnapaiyan, Hoshang J. Unwalla

**Affiliations:** Department of Immunology, Herbert Wertheim College of Medicine, Florida International UniversityMiami, FL, USA

**Keywords:** HIV, tobacco smoke, cocaine, mucociliary clearance, marijuana abuse, cystic fibrosis transmembrane conductance regulator, ciliary beat frequency

## Abstract

Impaired mucociliary clearance (MCC) is a hallmark of acquired chronic airway diseases like chronic bronchitis associated with chronic obstructive pulmonary disease (COPD) and asthma. This manifests as microbial colonization of the lung consequently leading to recurrent respiratory infections. People living with HIV demonstrate increased incidence of these chronic airway diseases. Bacterial pneumonia continues to be an important comorbidity in people living with HIV even though anti-retroviral therapy has succeeded in restoring CD4+ cell counts. People living with HIV demonstrate increased microbial colonization of the lower airways. The microbial flora is similar to that observed in diseases like cystic fibrosis and COPD suggesting that mucociliary dysfunction could be a contributing factor to the increased incidence of chronic airway diseases in people living with HIV. The three principal components of the MCC apparatus are, a mucus layer, ciliary beating, and a periciliary airway surface liquid (ASL) layer that facilitates ciliary beating. Cystic fibrosis transmembrane conductance regulator (CFTR) plays a pivotal role in regulating the periciliary ASL. HIV proteins can suppress all the components of the MCC apparatus by increasing mucus secretion and suppressing CFTR function. This can decrease ASL height leading to suppressed ciliary beating. The effects of HIV on MCC are exacerbated when combined with other aggravating factors like smoking or inhaled substance abuse, which by themselves can suppress one or more components of the MCC system. This review discusses the pathophysiological mechanisms that lead to MCC suppression in people living with HIV who also smoke tobacco or abuse illicit drugs.

## Introduction

With the introduction of combination antiretroviral therapy (cART) dramatic declines in morbidity and mortality from HIV/AIDS have been seen (Palella et al., 1998). HIV has become a treatable but chronic disease. People living with HIV live the lifespan equivalent to that of normal people. However, the incidence of respiratory diseases continues to plague the HIV-infected population. While a reconstituted immune response in these patients has decreased the incidence of opportunistic infections like Pneumocystis and bacterial pneumonia, HIV-infected individuals are still six times more likely to contract pneumonia compared to non-infected age matched controls (Sogaard et al., 2008). People living with HIV also present with increased incidence of chronic airway diseases like chronic bronchitis associated with COPD and asthma characteristically attributed to mucociliary dysfunction. The pathophysiology of these lung diseases in the context of HIV infections has still not been clearly understood. Recent studies show that the lower respiratory tract is a microbial reservoir in HIV-infected individuals rather than being a sterile environment as observed in healthy non-infected subjects and this may contribute to recurrent pneumonia and COPD in HIV-infected patients (Huang et al., 2011; Iwai et al., 2012). Moreover, the lung microbiome in People living with HIV is similar to that observed in COPD and cystic fibrosis (Huang and Lynch, 2011).

Impaired MCC, is primarily responsible for microbial colonization of airways in chronic airway diseases like COPD and cystic fibrosis (Sethi, 2000; Livraghi and Randell, 2007). MCC is a primary innate defense mechanism of the airways and protects the host from airborne pathogens, pollutants, and allergens (Wanner et al., 1996). Optimal MCC requires mucus, cilia, and a thin layer of ASL to facilitate ciliary beating. CFTR plays a pivotal role in maintaining ASL depth for optimal MCC by providing the necessary osmotic gradient through its ability to secrete Cl^−^ and enhancing paracellular permeability for fluid transport (Unwalla et al., 2015). Dysregulation of any component of the MCC system can attenuate MCC promoting microbial colonization. This results in chronic inflammation, progressive obstructive lung disease and recurrent lung infections.

People living with HIV demonstrate impaired nasal MCC (Kellerman, 2002; Robinson and Bye, 2002). Since the physiological mechanisms regulating nasal MCC are similar to tracheobronchial MCC it is possible that HIV suppresses this as well. HIV-infected individuals also abuse street drugs or smoke tobacco. Cigarette smoking or smoked substance abuse can exacerbate pulmonary disorders associated with HIV. We have demonstrated that TGF-β signaling, enhanced by cigarette smoke and in chronic airway diseases downregulates CFTR mRNA and function (Snodgrass et al., 2013; Unwalla et al., 2015) promoting mucociliary dysfunction and by consequence, microbial colonization. Smoked substance abuse involving marijuana and cocaine also act on the mucus and ciliary component of the MCC system. In this concise review we focus on the pathophysiological mechanisms by which HIV can by itself, or in combination with cigarette smoke or smoked substance abuse suppresses MCC.

## Mucociliary dysfunction of the airways

With inhalation of several thousand liters of air per day, human airway surfaces are constantly exposed to diverse environmental particles, allergens, and pathogens (Wanner et al., 1996). These agents are potent stimuli for airway inflammation and infections, if they are not removed efficiently from the lungs (Fujii et al., 2001; Gibson et al., 2003). Therefore, MCC has long been recognized as a primary innate defense mechanism of mammalian airways (barrier) that works in concert with a chemical shield of antimicrobial substances including lactoperoxidase, lysozyme, and lactoferrin, to protect the host from the noxious effects of airborne pathogens, pollutants, and allergens (Wanner et al., 1996; Ganz, 2002). The mucociliary apparatus consists of three functional compartments, that is, the cilia, a protective mucus layer, and an ASL layer in between the mucus and the ciliated cells to optimize ciliary beating. These mechanisms work in concert to remove inhaled particles from the lung.

Impaired MCC is directly responsible for productive cough, respiratory infection, and airflow obstruction observed in chronic airway diseases like cystic fibrosis and COPD associated with chronic bronchitis. Mucus transport is a function of ASL, ASL depth, and ciliary beating. Abnormalities in any compartment of the mucociliary system can compromise mucus clearance and cause chronic airway disease. Inability to clear mucus or excessive mucus secretion leads to microbial entrapment and promotes chronic infection (Gibson et al., 2003).

Ciliary beat frequency (CBF) can directly regulate MCC and this is evident in diseases like primary ciliary dyskinesia where attenuated ciliary beating leads to cough, infection, and airway obstruction (Afzelius, 1976, 1995). Ciliated cells are terminally differentiated columnar cells and their primary function in the epithelium is to propel the mucus toward the oral cavity by coordinated ciliary beating where it can either be expectorated or swallowed. These cilia are directly attached to the cell surface by the basal body. The baseline CBF in the upper airway is anywhere between 12 and 15 Hz (Fahy and Dickey, 2010). Based on external stimuli, the CBF can be increased or decreased by a number of signaling mechanisms (Salathe, 2007). While the precise mechanism by which CBF is regulated remains unknown a number of reports have demonstrated that phosphorylation of the dynein light chain by cAMP dependent Protein Kinase A (PKA) leads to increases in CBF (Salathe, 2007). Pharmacological drugs that lead to activation of adenylate cyclases or inhibit phosphodiesterases increase cAMP and lead to activation of PKA and consequently increase CBF (Lafortuna and Fazio, 1984; Wanner, 1985; Devalia et al., 1992; Milara et al., 2012; Unwalla et al., 2015). It has been suggested that changes in intracellular calcium [Ca]_i_ also increases CBF possibly by soluble adenylate cyclase mediated activation of PKA (Schmid et al., 2007).

Ciliary beating is also affected by periciliary ASL depth that allows cilia to beat efficiently and is crucial for mediating MCC rates (Boucher, 2002). Under normal conditions, the height of ASL is tightly regulated (Boucher, 2003). If the ASL depth is too high, the cilia cannot efficiently propel mucus. Conversely, unregulated ASL absorption (as seen in cystic fibrosis) results in ASL height reduction, impairing effective ciliary beating and leading to mucus impaction. Studies show that ASL autoregulation is associated with CFTR mediated inhibition of Na^+^ absorption and activation of Cl^−^ secretion (Boucher, 2003). Water follows through the transcellular/paracellular pathway maintaining ASL height leading to efficient MCC (Quinton, 1990; Tarran et al., 2001). While bronchial epithelial cells express aquaporins, deletion mutants of aquaporins demonstrate regular ASL depth pointing to a paracellular component for fluid transport (Verkman, 2007). It is now confirmed by a number of reports, including ours that CFTR also regulates the paracellular permeability of bronchial epithelium (Divac et al., 2004; Nilsson et al., 2010; Unwalla et al., 2015), placing CFTR at a critical juncture in ASL depth regulation where it not the necessary osmotic gradient but also paracellular permeability and thus fluid transport. In normal conditions, apical nucleotides (ATP and its metabolites) are important for hydrating airway surfaces (e.g., Tarran et al., 2006). ATP binds to purinergic G-protein coupled receptors leading to activation of Ca^2+^ dependent Cl^−^ channels and also CFTR. In chronic airway diseases like cystic fibrosis and COPD, CFTR function is either attenuated or absent. This leads to a significant decrease in epithelial Cl^−^ secretion, excessive Na^+^ absorption (Matsui et al., 1998) and a decreased paracellular permeability (Divac et al., 2004; Nilsson et al., 2010) an effect mimicked in COPD and chronic bronchitis (Divac et al., 2004; Kreindler et al., 2005; Cantin et al., 2006; Savitski et al., 2009) although not to the same extent. However, the system fully fails with additional insults (such as inflammation, which is more evident during disease exacerbations) resulting in reduced ASL depth mimicking cystic fibrosis.

Both, ASL depth maintenance (by CFTR function) and CBF activation rely on the common adenylate cyclase/cAMP/PKA pathway for activation making this pathway critical in maintenance of optimal MCC. HIV infection, environmental stimuli/pollutants like cigarette smoke and smoked substance abuse like crack cocaine, marijuana, methamphetamine can affect one or more components of the MCC system facilitating microbial colonization leading to recurrent lung infections and chronic airway diseases.

## HIV infection

HIV-infected individuals demonstrate all phenotypes of obstructive lung disease including small airways abnormalities, bronchiolitis, increases in airway obstruction, air-trapping, chronic bronchitis, deficits in DL_CO_ (Diffusing capacity of the lung for carbon monoxide), and anatomic and radiographic emphysema (Wallace et al., 1997; Diaz et al., 2000, 2003). HIV is an independent risk factor for COPD when compensated for smoking (Diaz et al., 2000). HIV-infected subjects are still six times more predisposed to contracting bacterial pneumonia compared to non-infected age matched controls in the post-cART era (Sogaard et al., 2008). Mortality, following an episode of bacterial pneumonia was also four times higher in HIV infected subjects compared to non-infected controls (Hirschtick et al., 1995). Indeed, around 10% of the causes of severe morbidity and 5% of the causes of death are related to pneumonia in industrialized countries (Bonnet et al., 2007; Hessamfar-Bonarek et al., 2010).

**Figure 1:**
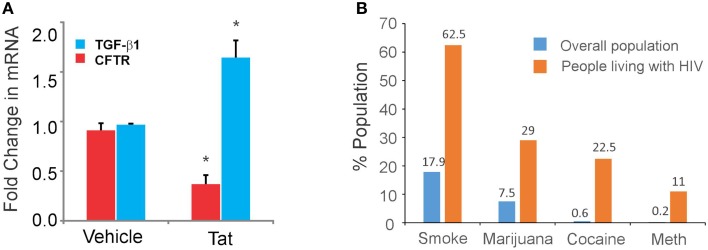
**(A)** HIV Tat induces expression of TGF-β1 mRNA with a concomitant decrease in CFTR mRNA levels. NHBE ALI cultures re-differentiated at the air liquid interface were treated with recombinant Clade B Tat (10 nM: ^*^p < 0.05) apically and basolaterally. Total RNA was isolated and TGF-β1 and CFTR mRNA levels were quantitated by qRT-PCR. HIV Tat induces almost a 1.7-fold increase in TGF-β1 mRNA expression. This translates to a significant decrease in CFTR mRNA levels. Data are mean ± SE of three experiments from three different lungs. **(B)** Nationwide trends in Cigarette smoking and drug abuse in general population compared with people living with HIV. While the trend in addiction is similar between the two, the proportion of people living with HIV who smoke cigarettes or abuse street drugs is significantly than that observed in the general population. Cigarette smoking is the most prevalent addiction [62.5% smokers (Benard et al., 2007; Lifson et al., 2010; Lifson and Lando, 2012) compared to 17.8% nationwide], followed by Marijuana [29% (Woolridge et al., 2005) compared to 7.5% nationwide], Cocaine [22.5% (Hinkin et al., 2004) compared to 0.6% nationwide] and methamphetamine [11% (Mitchell et al., 2006) compared to 0.2% nationwide]. The data for substance abuse in the general population is obtained from http://www.drugabuse.gov/national-survey-drug-use-health.

This could be due to attenuated MCC that promotes microbial colonization of the airways characteristically seen in chronic airway diseases with impaired MCC. HIV-infected individuals demonstrate abnormalities in the MCC apparatus (Kellerman, 2002; Robinson and Bye, 2002). While these studies have mainly dealt with nasal MCC, nasal Cl^−^ efflux, and CBF is often measured as a barometer of overall airway MCC health (Rutland et al., 1982; Cantin et al., 2006; Zhang et al., 2014). Moreover, there is an increased incidence of bronchiectasis, which is characterized by impaired MCC and recurrent infections, in People living with HIV (Holmes et al., 1992; Sheikh et al., 1997). Infected alveolar macrophages or other immune cells recruited by persistent inflammation (due to cigarette smoke, substance abuse, recurrent pneumonia, or other chronic airway diseases) can serve as reservoirs of HIV infection in the airway. While cART can control *de novo* infection and replication, viral proteins can still be expressed and secreted by these cells. Moreover, reports have convincingly shown that active HIV replication persists in infected individuals despite suppressive cART (Buzón et al., 2010; Hatano et al., 2013). Specifically, Tat expression is not suppressed by anti-retrovirals (Wu and Marsh, 2001, 2003; Kelly et al., 2008; Ensoli et al., 2010). Thus, infected immune cells can serve as a source of HIV proteins in the airway. Recurrent lung infections and other chronic inflammation associated with cigarette smoke can lead to recruitment of infected immune cells. While most clinical studies have reported undetectable levels of HIV in patients on cART, these studies involve rigorous follow-up by research coordinators to minimize the incidence of missed doses. Studies have shown that non-adherance rates vary widely from 33 to 75% (Knobel et al., 2009; Murphy et al., 2012). Missed doses or episodes of inflammation can lead to bursts of HIV replication and increase viral proteins in the lung. HIV Tat protein has a protein transduction domain that allows its secretion by infected cells and uptake by bystander cells where it mediates pleotropic effects (Frankel and Pabo, 1988; Ensoli et al., 1990, 1993; Chang et al., 1997). We have already demonstrated that TGF-β1 signaling increased in chronic airway diseases and in smokers can suppress CFTR function (Unwalla et al., 2015). HIV Tat has been shown to induce TGF-β1 expression in a number of cell types (Gibellini et al., 1994; Thatikunta et al., 1997; Reinhold et al., 1999) possibly by binding to a Tat responsive element in the TGF-β1 promoter (Cupp et al., 1993; Thatikunta et al., 1997). Recombinant HIV Tat increases TGF-β1 mRNA in primary human bronchial epithelial cells and this leads to a concomitant decrease in CFTR mRNA (Figure 1A and Unwalla, 2015).

Although mucus is an essential component of MCC, excessive mucus production can contribute to airway obstruction and pathogenesis of COPD, airway inflammation, asthma, and chronic bronchitis. HIV env protein gp120 has been shown to increase mucus production in primary bronchial epithelial cells. Gundavarapu et al. have demonstrated that gp120 derived from X4 tropic viral strains but not R5 tropic viral strains increase mucus production in differentiated primary human bronchial epithelial cells (Gundavarapu et al., 2013). Inhibitors of CXCR4 and α7-nAChR-GABA_A_Rα2 blocked mucus production in differentiated primary human bronchial epithelial cells *in vitro* suggesting that HIV gp120 signals by binding to CXCR4 receptor and this pathway also involves α7-nAChR-GABA_A_Rα2. A number of reports have suggested that binding of HIV gp120 to its co-receptors results in signaling cascades that facilitate viral entry and replication (Stantchev and Broder, 2001; Freedman et al., 2003; Yi et al., 2004). CXCR4 is a G-protein coupled receptor that associates with Gαi which inhibits adenylate cyclase activation. Since both CFTR activation and ciliary beating depends on the Adenylate cyclase/cAMP/PKA pathway, gp120 can potentially suppress CFTR activation as well. Thus, HIV proteins Tat and gp120 can by itself suppress MCC in the airways. Approximately 80% of HIV-infected individuals also smoke tobacco or abuse other street drugs (NSDUH, 2010 and Figure 1). Mucociliary dysfunction can be exacerbated in People living with HIV who smoke tobacco or street drugs since smoking and drug abuse are independent risk factors for mucociliary dysfunction often presenting with airway diseases characteristic of impaired mucociliary function like chronic bronchitis associated with COPD, asthma, and recurrent lung infections.

## Smoked substance abuse

Despite the declining prevalence of smoking in the United States (http://www.cdc.gov/tobacco/data_statistics/tables/trends/cig_smoking/index.htm), a significant proportion of HIV-infected individuals are cigarette smokers or smoked substance abusers (Figure 1B). Indeed the proportion of people who smoke tobacco or other illicit drugs is significantly higher in people living with HIV compared to trends observed in overall population (http://www.drugabuse.gov/national-survey-drug-use-health). Almost 60% of People living with HIV are also smokers (Benard et al., 2007; Lifson et al., 2010; Lifson and Lando, 2012). Cigarette smoking is the most prevalent addiction followed by Marijuana, Cocaine and methamphetamine. Cigarette smoking and smoking street drugs result in the airway exposed to the highest concentration of these drugs. Use of methamphetamine by smoking is the fastest growing mode of administration, which increases concerns about potential pulmonary and other medical complications. Currently, no peer-reviewed papers exist that have investigated the effects of methamphetamine abuse on the mucociliary system. Cigarette smoke by itself is a potent risk factor for chronic bronchitis associated with COPD. Chronic bronchitis, even though a clinical diagnosis, is characterized by mucus hypersecretion and reduced MCC. Cigarette smoke can suppress MCC by directly interfering with all three components of the MCC apparatus namely, increasing mucus secretion (Mebratu et al., 2011), reducing CBF as well as shortening cilia length (Cohen et al., 2009; Leopold et al., 2009) and suppressing ASL depth by inhibiting CFTR either directly, by sequestering surface CFTR molecules in aggregosomes and or by suppressing CFTR biogenesis via TGF-β signaling (Cohen et al., 2009; Clunes et al., 2012; Unwalla et al., 2015). While Cigarette smoke only activates available TGF-β1 to suppress CFTR biogenesis, it does not increase in TGF-β1 levels in airway epithelial cells (Unwalla et al., 2015). HIV Tat on the other hand also increases TGF-β1 mRNA levels and/or signaling. Thus, in HIV infected patients there is increased availability of TGF-β1. Hence in HIV infected smokers CFTR suppression can be exacerbated due to an additive effect of HIV Tat and cigarette smoke. This can decrease the periciliary fluid leading to attenuated ciliary beating. Moreover, HIV gp120 can also stimulates mucus hypersecretion (Gundavarapu et al., 2013). Hence it is expected that a combination of HIV and smoking can lead to a profound suppressive effect on MCC.

Likewise Marijuana smoking can also synergize with HIV infection to have an additive effect on MCC suppression. While short-term marijuana use has not been implicated in any decrease in pulmonary function, when compared to tobacco smoke, long term cannabis smoking results in symptoms similar to that observed in smokers with coughing, chronic bronchitis and increased mucus production. While there are no reports of any direct or indirect action of marijuana smoking on CFTR function, marijuana smoking has been shown to decrease ciliated cells, increase mucus-producing cells and lead to cellular disorganization with squamous metaplasia (Gong et al., 1987). In HIV-infected marijuana smokers, a combination of factors like CFTR mRNA suppression (by Tat), increased mucus production (due to gp120 and effects of marijuana) and decreased number of ciliated cells (by marijuana) can lead to MCC suppression greater than that observed for marijuana or HIV alone.

Cocaine abuse either by way of snorting crystalline cocaine or smoking crack cocaine results in the airway exposed to the highest concentration of this drug. Asthma and COPD are common among cocaine users (Rubin and Neugarten, 1990; Leece et al., 2013). Moreover, a single exposure to cocaine can lead to persistence of the drug in the airway hours after smoking (Byck and Van Dyke, 1977). Cocaine has been shown to decrease CBF with higher doses causing irreversible ciliostasis (Barton and Gray, 1979; Robson et al., 1992; Ingels et al., 1994). Apart from its effects on ciliary beating, Cocaine has also been shown to affect the airway mucosa in several other ways. Cocaine, at high concentrations has been shown to suppress basal short circuit current (generally a function of Cl^−^ efflux or Na^+^ absorption from the mucosal side of the epithelium). Cocaine was also shown to suppress Cl^−^ efflux in response to CFTR potentiators like β_2_-agonsits (Farley et al., 1991). In HIV-infected individuals that also abuse crack cocaine, the combined effects of Tat-mediated suppression of CFTR (and by extension ASL depth) and cocaine mediated ciliary dyskinesia or ciliostasis can lead to a synergistic effect on MCC suppression.

## Restoration of MCC in people living with HIV who also abuse street drugs

**Figure 2:**
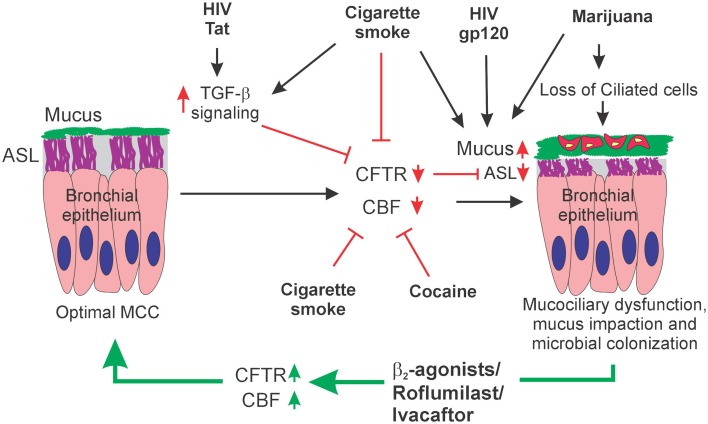
**Schematic model of HIV and substance abuse induced Mucociliary dysfunction**. HIV Tat and Cigarette smoke can inhibit CFTR biogenesis and function. HIV Tat increases TGF-β1 mRNA levels with a corresponding decrease in CFTR mRNA. Cigarette smoke can suppress CFTR biogenesis by TGF-β1 signaling or directly inhibit CFTR function by trapping surface CFTR in aggregosomes. Cigarette smoke, marijuana and cocaine can also inhibit the Ciliary component of MCC by decreasing CBF or ciliostasis (by cocaine). Marijuana smoking can lead to a loss of ciliated cells in the airway epithelium. The effects of these drugs on the ciliary component can synergize with the effects of HIV Tat mediated suppression of CFTR leading to a pronounced suppression of MCC. A combination of HIV gp120, Cigarette smoke and/or Marijuana can also promote mucus hypersecretion in the milieu where CFTR function is already attenuated by Tat and cigarette smoke leading to Dysregulation of all the principal components of the MCC system. Dysregulation of one or more components of the MCC system will lead to mucus impaction and microbial colonization. Pharmaceutical drugs that increase intracellular cAMP by either activating cyclase or inhibiting phosphodiesterase when used in combination with CFTR potentiators like Ivacaftor can restore one or more components of the MCC and repair the mucociliary dysfunction.

Two major components of the MCC apparatus namely, ASL depth maintenance (as a consequence of CFTR function) and CBF depend on the adenylate cyclase/cAMP/PKA pathway. Hence therapeutics that potentiate this pathway can be used to restore MCC in HIV infected individuals and/or smoked substance abusers (Figure 2). β_2_-adrenergic receptors are spatially and functionally coupled to the adenylate cyclase/cAMP/PKA pathway and CFTR (Naren et al., 2003). The use of β_2_-agonists as an alternative mechanism to restore MCC along with its prescribed use as bronchodilators in chronic airway diseases like asthma and COPD is attractive in that a time-tested drug can serve a dual purpose to ameliorate two symptoms associated with these diseases. Apart from their known ability to serve as bronchodilators, we and others have shown that they can potentially improve MCC in three distinct ways by increasing CBF (van As, 1974; Wanner et al., 1996; Unwalla et al., 2015), activating CFTR (Gilljam et al., 1987; Unwalla et al., 2015), and consequently, increasing paracellular permeability (Unwalla et al., 2012). Alternately, another drug Roflumilast, a Cyclic nucleotide phosphodiesterase (PDE) inhibitor can strongly increase Cl^−^ efflux by CFTR, enhance CBF, and MCC in COPD and in response to cigarette smoke (Baumlin et al., 2014; Lambert et al., 2014; Tyrrell et al., 2015). The cAMP-selective PDE4 family is a major isoform found in respiratory epithelia and in resident immune cells of the lung. Inhibition of PDE4 would increase cAMP available for PKA mediated activation of CFTR and CBF. A synergistic effect is observed on MCC restoration when Roflumilast is used in combination with β_2_-agonists (Baumlin et al., 2014). This could be because β_2_-agonsists enhance cAMP production by Gαs mediated activation of adenylate cyclase while PDE inhibition by Roflumilast decreases the turnover of cAMP. Alternately CFTR potentiators like Ivacaftor can also been used to restore CFTR function and enhance MCC (Sloane et al., 2012).

## Conclusions

Normal mucociliary function fails with dysfunction of any one of the major components of the MCC apparatus namely mucus production, ciliary beating, and ASL depth maintenance fail. Suppression of MCC leads to an inefficient clearance of pollutants, pathogens, and allergens. This leads to chronic inflammation and microbial colonization which manifests as chronic airway diseases like asthma, chronic bronchitis associated with COPD, and recurrent lung infections pervasive in HIV infected individuals and/or smoked substance abusers. Smoked substance abuse can suppress MCC independently and an underlying HIV infection can have an additive effect as each of these can suppress one or more components of MCC system. Under these conditions, therapeutics that can restore CFTR function or CBF can restore MCC and prevent microbial colonization consequently decreasing the incidence of pneumonia in People living with HIV.

### Conflict of interest statement

The authors declare that the research was conducted in the absence of any commercial or financial relationships that could be construed as a potential conflict of interest.
